# Inherited Neuromuscular Disorders: Which Role for Serum Biomarkers?

**DOI:** 10.3390/brainsci11030398

**Published:** 2021-03-21

**Authors:** Antonino Lupica, Vincenzo Di Stefano, Andrea Gagliardo, Salvatore Iacono, Antonia Pignolo, Salvatore Ferlisi, Angelo Torrente, Sonia Pagano, Massimo Gangitano, Filippo Brighina

**Affiliations:** Department of Biomedicine, Neuroscience and advanced Diagnostic (BIND), University of Palermo, 90121 Palermo, Italy; vincenzo19689@gmail.com (V.D.S.); andrigl@gmail.com (A.G.); salvo.iak@gmail.com (S.I.); niettapignolo@gmail.com (A.P.); fransalvo1@gmail.com (S.F.); angelotorrente92@gmail.com (A.T.); sonia.pagano@policlinico.pa.it (S.P.); massimo.gangitano@unipa.it (M.G.); filippobrighina@gmail.com (F.B.)

**Keywords:** biomarkers, inherited neuromuscular disorders, rare diseases

## Abstract

Inherited neuromuscular disorders (INMD) are a heterogeneous group of rare diseases that involve muscles, motor neurons, peripheral nerves or the neuromuscular junction. Several different lab abnormalities have been linked to INMD: sometimes they are typical of the disorder, but they usually appear to be less specific. Sometimes serum biomarkers can point out abnormalities in presymtomatic or otherwise asymptomatic patients (e.g., carriers). More often a biomarker of INMD is evaluated by multiple clinicians other than expert in NMD before the diagnosis, because of the multisystemic involvement in INMD. The authors performed a literature search on biomarkers in inherited neuromuscular disorders to provide a practical approach to the diagnosis and the correct management of INMD. A considerable number of biomarkers have been reported that support the diagnosis of INMD, but the role of an expert clinician is crucial. Hence, the complete knowledge of such abnormalities can accelerate the diagnostic workup supporting the referral to specialists in neuromuscular disorders.

## 1. Background and Aims

Inherited neuromuscular disorders (INMD) are rare diseases that involve muscles, motor neurons, peripheral nerves or the neuromuscular junction [1].

Even if the most important role in the diagnosis of INMD is played by clinical and subsequent examinations (e.g., neurophysiological, histo-morphological and genetic investigations), serum biomarkers may have a crucial role in the diagnosis and follow-up.

Indeed, it is a well-known fact that serum biomarkers have a crucial role in the diagnosis of many acquired neuromuscular conditions ranging from autoimmune, inflammatory and paraneoplastic diseases affecting the muscle and peripheral nerve [2,3,4], but their role appears to be less immediate and appreciated for INMD [1].

A particular mentioning for genetic investigations is worth in the diagnostic workup: indeed genetics is in rapid evolution and has radically modified the approach to rare diseases in recent years [1,5,6]. In particular, the recent availability of next generation sequencing has deeply revolutioned the scenario of INMD, compared to only a few years ago, when the diagnostic algorithms were based on the sequencing gene by gene (e.g., Sanger), based on clinical, neurophysiology, biochemical and morphological evaluations [1]. In this scenario, serum biomarkers retain an important role in both the diagnosis and follow-up of INMD [7,8,9,10]. Many lab abnormalities commonly evaluated in routine blood examinations have been reported “typical” of certain INMD [8,11,12], but their role is sometimes underestimated in current clinical practice [13]. Moreover, lab samples are usually cheap, easy-to-obtain and, in many circumstances, they are parts of other general clinical procedures. Conversely, some biomarkers are very unspecific (e.g., serum creatine kinase (CKO), but associated conditions may point out the underlining condition (e.g., concomitant factors; the entity and/or the persistence of the abnormalities) [8,14] or rule out other differential diagnoses [15]. Finally, in specific clinical conditions, some biomarkers commonly used in clinical practice are indicative only if the sample is collected in a specific condition [16].

Moreover, the correct use of biomarkers in INMD allows a specific time- and resource-sparing algorithm. In fact, INMD patients, if promptly evaluated through biomarkers, can reach the correct diagnosis earlier and receive consequently an adequate management and therapy [17,18]. Occasionally, lab essays are abnormal despite the patient does not display any clinical sign (e.g., carriers); in these cases, a serum abnormality encourages the beginning of a diagnostic workup allowing a prompt genetic counselling [10,19].

Multiple organs are usually involved in INMD, so that patients are often evaluated by multiple clinicians other than expert in NMD before the diagnosis [20,21].

In this paper, we present a review of serum biomarkers in INMD to provide a guide to address the diagnosis and the correct management. INMD have been classified depending on the main organ involved (i.e., spinal cord, peripheral nerve, muscle, etc.) and separately analyzed as neuropathies (axonal and demyelinating), neuromuscular junction’s disorders, myopathies (muscular dystrophies, Ion channel myopathies, metabolic myopathies) and hereditary motor neurons diseases.

## 2. Muscular Dystrophies

Muscular dystrophies (MD) constitute a heterogeneous group of INMD with progressive muscle weakness. Duchenne muscular dystrophy (DMD) is the most common and most severe MD and it is characterized by a complete lack of dystrophin due to mutations in the DMD gene [19]. Serum creatine kinase (CK) concentration as well as transminases (Supplementary Materials: Table S1 and Diagram S1) are markedly increased in children with DMD prior to the appearance of any clinical sign of disease; increased values are even observed among newborns [22]. Of interest, the CK peak by age of two years is usually from 10 to 20 times the upper normal limit, even if higher values have been reported [23]; serum CK progressively fall about 25% per year, eventually reaching the normal range in most cases, as the skeleton muscle is replaced by fat and fibrosis. In patients with Becker muscular dystrophy (BMD), CK concentration is usually elevated above five or more times the normal limits [19]. Serum CK is usually modestly elevated in limb-girdle muscular dystrophy (LGMD). However, it can be very high in sarcoglycanopathy, dysferlinopathy, and caveolinopathy [24]. Of interest, in LGMD2L, a condition resulting from mutations in ANO5, the gene for anoctamin 5, CK are usually elevated to around 1500 to 4500 U/L (range 200–40,000 U/L) and tend to decrease over time [24]. Furthermore, in DMD patients carbonic anhydrase 3 (CAH3), microtubule-associated-protein-4 and collagen type I-alpha 1 chain concentrations decline constantly over time; of interest, myosin light chain 3 (MLC3), electron transfer flavoprotein A (ETFA), troponin T, malate dehydrogenase 2 (MDH2), lactate dehydrogenase B and nestin plateaus in early teens; ETFA correlates with the score of the 6-min-walking-test, whereas MDH2, MLC3, CAH3 and nestin correlate with respiratory capacity [25]. In addition, high serum concentrations of a set of muscle-enriched miRNAs, (miR-1, miR-133, miR-206, miR-208b, miR-208a and miR-499) were significantly elevated in DMD patients compared to healthy subjects [7,18].

Serum matrix metalloproteinase-9 (MMP-9) and its inhibitor (TIMP-1) have been proposed as markers of disease progression in DMD [26]. In DMD patients the decrease in creatinine and an increase in creatine serum concentrations is likely due to the insufficient creatine utilization by muscles. Similar profiles have been observed in other MD such as BMD, LGMD2A and LGMD2B. Creatine/creatinine ratio is particularly elevated in the older and more severely affected DMD patients, thus encouraging its use as a marker of disease progression. Reduced citrulline concentrations have been found in DMD patients [27]. There is a significant increase in fibronectin concentrations in DMD patients compared to age-matched controls, while a similar abnormality is not observable in BMD [28].

Patients with myotonic dystrophy type 1 and 2 (DM1 and 2) commonly present modestly elevated serum CK (less than 500 U/L) [29]. Low concentrations of anti-Müllerian hormone (AMH) demonstrates decreased ovarian reserve in women with DM1 [30]. Results of a study in a cohort of DM patients [14] showed a persistent elevation of high sensitivity cardiac troponin T (hs-cTnT) and CK; in contrast, high sensitivity cardiac troponin I (hs-cTnI) values were persistently normal throughout the study. In addition, a relevant quote of DM patients with cardiac conduction abnormalities and preserved systolic function presented abnormal NT-proBNP concentration [14]. DM1 patients had insulin-resistance and significantly higher triglycerides (TGs), glucose and Tumor Necrosis Factor A; conversely, they had significantly lower concentrations of total serum adiponectin with a selective, pronounced decrease of its high molecular weight oligomers [31]. Finally, sleep dysfunction, very common in DM1, may be mediated by a dysfunction of the hypothalamic hypocretin because of hypocretin1 reduction in CSF [32].

Facioscapulohumeral muscular dystrophy (FSHD), the third most common type of MD, is characterized by slowly progressive muscle weakness involving the facial, scapular, upper arm, lower leg and hip girdle muscles, usually with asymmetric involvement. Serum CK can be normal or modestly elevated in FSHD (usually less than five times the upper normal limit) [33]. Finally, an elevation of serum muscles (MM) and myocardial band (MB) isoforms of CK, as well as carbonic anhydrase III, and troponin I type 2 have a minor role to assess the severity and progression of FSHD [8].

In Emery-Dreifuss muscular dystrophy (EDMD), a modest elevation of serum CK concentration is typical, less than 1000 U/L; an increase up to 20 times the upper normal limit sometimes occurs, but it may be seen often higher in the early stages of the disease [34]. The concentrations of circulating tenascin-C (TN-C) are elevated in autosomic dominant-EDMD and X-linked-EDMD patients and in some X-linked-EDMD carriers, allowing an identification of EDMD patients at risk of dilated cardiomyopathy [35]. Transforming growth factor beta 2 (TGF b2) and interleukin 17 (IL-17) serum values are consistently elevated in EDMD type 2 and LGMD1B patients [36].

GNE myopathy, where “GNE” is an abbreviation for the mutated gene (*UDP-N-acetylglucosamine 2-epimerase/N-acetylmannosamine kinase)* codifying the key enzyme of sialic acid synthesis, is an adult-onset progressive myopathy, in which a mild serum CK elevation is reported [37]. The ratio of the Thomsen–Friedenreich (T)-antigen to its sialylated form, ST-antigen, detected by liquid chromatography—mass spectrometry and liquid chromatography—tandem mass spectrometry (LC-MS/MS), is a robust blood-based (serum or plasma) biomarker informative for diagnosis and possibly for response to therapy for this rare condition [38].

## 3. Metabolic Myopathies

Metabolic myopathies are INMD in which the pathogenetic mechanism compromises enzymes and proteins involved in intermediary metabolism of glucose and free fatty acids [16]. In this review, we focused on glycogenoses, disorders of muscular lipids metabolism and mitochondrial myopathies.

### 3.1. Glycogenoses

Muscle glycogenoses constitute a growing number of inborn errors of glycogen metabolism. Deficiencies of virtually all enzymes, which intervene in the synthesis or degradation of glycogen, may cause glycogen storage disease (GSD) because of aberrant storage or utilization of glycogen [39].

In this group McArdle and Pompe disease are the most common conditions [40,41]. CK is chronically elevated in nearly all cases of McArdle disease; patients can often show rabdomyolisis and hemoglobinuria after a short-intensity exercise, depicting a very typic feature of this disorder [16]. Apart from CK, serum lactate after forearm exercise is another simple tool that can be very useful to early recognize this disorder [42]. Patients with the most common McArdle disease mutation (R49X) have no protein expressed because of nonsense-mediated mRNA decay; consequently, they have a secondary deficiency of pyridoxine (vitamin B6), due to protein-to-protein interaction [16,43].

Other metabolic disorders such as Pompe disease (PD) present a less specific pattern due to different biochemical pathway involved [44]. HyperCKemia is usually found in PD, but the increment of CK is stably high and a muscular stretching impairment is often observed mimicking a LGMD [41]; moreover, dried blood spot offers an important tool to screen suspected patients for PD [45,46].

Further serum biomarkers have a less validated use in clinical practice: some authors reported that platelet derived grow factor BB (PDGF-BB) and transforming growth factor β1 (TGF-β1) concentrations are significantly lower in PD patients compared to the control group and serum; conversely, platelet derived grow factor AA (PDGF-AA) and connective tissue growth factor (CTGF) values are significantly higher compared to control samples. Interestingly, PDGF-BB level differs among PD and MD and between symptomatic and asymptomatic PD patients [47].

In addition, GSD can be associated with hemolysis/hemolytic anemia (e.g., GSD7, phosphoglycerate kinase 1 deficiency, GSD12). Finally, many glycogenolytic and glycolytic defects have been also linked to myogenic hyperuricemia, which can lead to gout [48].

### 3.2. Disorders of Muscular Lipids Metabolism

Disorders of muscular lipids metabolism may involve intramyocellular triglyceride degradation, carnitine uptake, long-chain fatty acids mitochondrial transport, or fatty acid β-oxidation [17]. In these conditions, blood testing for CK, lactate, and glucose is usually normal between the episodes of rhabdomyolysis [17]. However, during an acute bout of rhabdomyolysis, a significant increase of CK starts within a few hours, during which hyperkalemia and hypoketotic hypoglycemia can also occur [17]. Moreover, in more severe cases, an acute renal failure can occur with elevations of potassium, creatinine, and urea [17]. The acylcarnitine profile, performed by liquid chromatography tandem mass spectrometry, is the most sensitive and specific test for a fatty acid oxidation defects [16].

Carnitine deficiency (CD) is a potentially lethal but very treatable disorder due to a defect in the carnitine organication transporter (OCTN2), resulting in impaired fatty acid oxidation [49].

CD is clinically characterized by carnitine-responsive acute metabolic decompensation early in life, or in late onset forms, with skeletal and cardiac myopathy or sudden death from arrhythmia [50,51,52].

Hypoglycemia, with minimal or no ketones in urine, and hyperammonemia, with variably elevated liver function tests are typical features of this disorder [53].

The deficiency of carnitine–palmitoyl-transferase-II (CPT-II, an enzyme involved in the transport of fatty acids into the mitochondrial matrix) is clinically characterized by recurrent myoglobinuric attacks triggered by exercise, fasting, fever, and infection, which may be complicated by acute renal failure and respiratory failure [49].

Multiple acyl-CoA dehydrogenation deficiency (MADD) is a disorder of oxidative metabolism with a broad range of clinical severity [49]; symptoms and age of onset are highly variable and characterized by muscle involvement (myalgia and weakness recurrent episodes of lethargy, vomiting, hypoglycemia, metabolic acidosis, and hepatomegaly) [54].

The diagnosis is suggested by the acyl-carnitine profile and urinary organic acids, revealing low serum free carnitine but elevated acyl-carnitines [49].

Neutral lipid storage disease with myopathy (NLSD-M), increased CK concentrations, cardiomyopathy, diabetes mellitus, hepatic steatosis, and hypertriglyceridemia. Leukocytes show a characteristic accumulation in cytoplasm of triglycerides called “Jordan’s anomaly” [49].

### 3.3. Mitochondrial Myopathies

Mitochondrial myopathies (MM) are disorders with mitochondrial function impairment, which usually present multisystem features and, sometimes specifical biomarkers have been recognized [55].

Elevated serum basal lactate have a sensitivity of approximately 65% and specificity of 90% [56], the abnormal lactate value is more evident after aerobic exercise test, an important tool in the clinical suspected of MM [55].

Serum CK is often normal in MM, but it might result elevated after rhabdomyolysis and in a minority of patients [55]. CK concentrations continually higher than three times the normal upper limit should arise a prompt consideration of a different diagnosis [16].

About 50% of patients with Mitochondrial encephalopathy with lactic acidosis and stroke-like episodes (MELAS) can manifest with type 2 diabetes mellitus or impaired oral glucose tolerance [16,57,58]. Plasma amino acid testing can reveal elevated alanine, especially after aerobic exercise. Urine organic acid analysis may reveal high 3-methylglutaconic acid or the tricarboxylic acid intermediates (fumarate, malate, citrate) [16]. Increased CSF proteins, lactic acidemia and hyperalaninemia are also common in mitochondrial neurogastrointestinal encephalopathy [59], an autosomal recessive condition due to mutations in the gene specifying thymidine phosphorylase with progressive gastrointestinal symptoms and muscle involvement [60]. The diagnosis is based on the detection of pathogenic mutation, reduction of thymidine phosphorylase enzyme activity or elevated plasmatic thymidine and deoxyuridine.

Cycle ergometry exercise testing in mitochondrial myopathies might demonstrate a low the maximum oxygen consumption a high respiratory exchange ratio (indicative of early lactate production), or both [16]. Moreover, the oxidative stress have a relevant pathogenic role in MM; hence, oxidative stress markers, such as advanced oxidation protein products have been successfully tested in MM [61].

## 4. Ion Channel Myopathies

Ion channel myopathies (ICM) include various rare INMD due to mutations in genes encoding ion channels Main disorders of this group are periodic paralyses (PP) and non-dystrophic myotonias (NDM) [62].

The diagnosis of such myopathies relies on the patient’s personal and familial medical history and several laboratory findings, with genetic studies representing the diagnostic mainstay.

As far as hypo- and hyperkaliemic periodic paralyses (PP) are concerned, serum diagnostic supportive criteria are constituted by K^+^ concentrations <3.5 and >4.5 mEq/L during weakness attacks, respectively [63,64,65,66,67,68,69,70,71,72,73]. Raised CK may be present too [74,75]. Moreover, thyroid function tests may be useful to distinguish HypoPP from thyrotoxic paralysis [76,77], because of HypoPP is reported as a rare complication of hypertyroidisim [78].

CK elevation is common in myotonia congenita [75,79,80,81,82,83,84,85,86,87] and may be present in paramyotonia congenita [88]. Inherited susceptibility to malignant hyperthermia syndrome (MHS) occurs in patients exposed to anesthetic agents causing increased body temperature, muscle rigidity or spasms, rhabdomyolysis, tachycardia, and other life-threatening symptoms [89]. Mutations in the ryanodine receptor RYR1, CACNA1S or STAC3 genes are encountered in MHS, resulting in excessive cytoplasmic calcium accumulation in response to both pharmacological and non-pharmacological triggers. As a result, patients experience recurrent episodes of hyperCKemia and rhabdomyolysis [11,89,90]. RYR1-related central core myopathy usually shows elevation of CK [91,92,93,94], and, in some cases, it can be also normal [95], AST, ALT and γGT may be elevated or normal [93,96].

## 5. Congenital Myasthenic Syndromes

Congenital myasthenic syndromes (CMS) refer to a heterogenic group of inherited neuromuscular transmission disorders (NTD) with exercise intolerance and muscular weakness [97]. There are no relevant biomarkers for CMS and the diagnosis is usually achieved through neurophysiology [97]. Indeed, CK concentrations are usually normal in CMS [97], but there are reported cases in which CK and aldolase concentrations can be mildly elevated [98,99]. In these cases, elevated CK is due to a phenomenon called endplate myopathy [99]. Circulating miRNAs [100] and plasmatic titin [101] have been proposed as possible biomarkers for autoimmune *Myasthenia Gravis*, but their role has not been explored yet in inherited CMS.

## 6. Motor Neurons Diseases

### 6.1. Spinal Muscular Atrophy

Spinal muscular atrophy (SMA) is characterized by motor neuron degeneration and progressive muscle atrophy and weakness caused by mutation of the survival motor neuron (*SMN*) genes. After neuronal degeneration or axonal injury specific cytoskeletal proteins of the neurons, including neurofilaments (NF), are released in the extracellular space, in Cerebrospinal fluid (CSF) and in the blood; about 80% of NF are phosphorylated (pNF) conferring more resistance in the serum. Several studies explored the role of NF as a biomarker of disease severity and progression. There are three different NF type distinguished by molecular weight: light (NF-L), medium and heavy (NF-H). High concentrations of NF-H have been detected in blood of amyotrophic lateral sclerosis, SMA, Alzheimer’s disease, Parkinson disease and multiple sclerosis [102,103,104,105,106,107].

Plasma phosphorylated neurofilament heavy chain (pNF-H) in infants with SMA are about 10-fold higher than age-matched infants without SMA. Treatment with nusinersen (the first approved treatment for SMA favoring expression of normal SMN protein) induce a faster decline in pNF-H concentrations at two and ten months than sham controlled-treated infants, followed by a relative stabilization [108]. These data suggest NF as a promising biomarker for SMA severity and response to treatment.

Patients with SMA have been shown to have lower values of creatinine compared to age-matched controls [109]. In the skeletal muscles is stored most creatine of the body, and its metabolism is essential to maintain the muscle function; creatinine is a product of muscle creatine metabolism. Chronic medical conditions associated with cachexia (and muscle hypotrophy) could be associated with lower concentrations of creatinine. A recent study [109] showed significant differences of creatinine concentrations depending on the SMA subtype (with SMA 3 > SMA 2 > SMA 1 concentrations) and on clinical severity; furthermore, a positive association of serum creatinine concentrations and the number of *SMN2* gene copies has been documented; a positive association of creatinine concentrations was also related to denervation signs expressed by maximal compound muscle action potential amplitude evoked by distal stimulation of ulnar nerve, and to the motor unit number estimation. Considering that the muscle atrophy could be considered a confounding factor for low creatinine concentrations, all the reported associations with the severity of disease, still resulted statistically significant, after correction for lean mass [109].

### 6.2. Amyotrophic Lateral Sclerosis

Amyotrophic lateral sclerosis (ALS) causes upper and lower motor neuron degeneration, with progressive paresis of arms, legs and bulbar muscles finally resulting in death for respiratory failure.

Most ALS cases are sporadic and only about 10% are familiar as associated to various specific gene mutations (e.g., *SOD1, TDB-43, FUS, DPRs*, etc.).

Even if an aspecific marker serum CK is reported in an high percentage of ALS patients [110], markers of inflammation [111,112] as well as creatinine [113] have a prognostic role in ALS.

The most studied biomarker for SLA is the pNF-H which show increased concentrations both in CSF (sensitivity 71%, specificity 88%) [114] and in the blood (sensitivity 58%, specificity 89%) [115]. As showed in SMA and other neurodegenerative diseases, the axonal injury induces the release of these cytoskeletal proteins in the extracellular space, CSF and blood. However, this measure is not specific for familiar ALS respect to sporadic forms and it is not statistically different in Riluzole-treated vs. non-treated patients [115]. By the way, NF still represent a possible marker for upcoming therapies.

Testing the hypothesis of ALS as an autoimmune pathology, neuron glycolipids such as sulfoglucuronosyl paragloboside (SGPG), target of various neuropathies, have been explored in ALS and anti-SGPG were found in the sera of 13.3% ALS patients [116].

Other biological markers of ALS remain of uncertain significance in the pathobiology of disease, but include transthyretin, complement C3 protein and fetuin-A [117,118,119].

## 7. Axonal Neuropathies

This group includes disorders in which polyneuropathy (sensory-motor deficit associated with reduced jerks in the distribution of the main peripheral nerves) is the main feature and disorders with nerve involvement as part of multisystemic disease.

### 7.1. Charcot–Marie–Tooth 2 (CMT2)

Charcot–Marie–Tooth (CMT) is an autosomal dominant inherited motor and sensory neuropathy, characterized by peripheral nerve damage and classically described in CMT Type 1 and 2 depending on the neurophysiological features [120]. CMT2 is genetically heterogeneous and exists in several different subtypes (e.g., CMT2A, CMT2B, CMT2E, etc.) [20]. CMT2 is an axonal form of the disease in which the loss of axons reduces muscle innervations [120]. Neurofilament light chain (NfL) represent a possible biomarker of axonal damage in CMT2 correlating with disease severity [121].

### 7.2. Multisystemic Diseases

The term “amyloidosis” gathers several diseases associated with deposits of amyloid fibrils involving peripheral and autonomic nervous systems; among them about 10% is represented by inherited forms (hereditary amyloidosis, HA) [122]. HA includes Hereditary amyloidogenic transthyretin (hATTRv) [123], amyloidosis derived from a fibrinogen variant (AFib), that is the most frequent HA in Northern Europe [124], variants of apolipoproteins (AI, AII, C-II, CIII), gelsolin (AGel), and lysozyme (ALys) [125].

In a study on hATTR, serum retinol-binding protein 4 (RBP4) concentration was lower in patients with hATTR V122I amyloidosis; also a lower concentration of B-type natriuretic peptide (BNP) and transtiretin (TTR), as well as, a higher concentration of Troponin I (TrI) and hematocrit have been reported [126]. Increased serum concentrations of amino-terminal prohormone of brain natriuretic peptide (NT-proBNP) and Troponin T (TrT) are the mainstay in patients with polineurophaty compared to *TTR*v carriers and healthy subjects [127]. Of interest, serum neurofilament light chain (NfL) concentrations correlated with Troponin T in all patient groups, but not with NT-proBNP. Furthermore, higher cytokine concentrations (TNF-α, IL-1β, IL-8, IL-33, IFN-β, IL-10 and IL-12) have been reported in hATTR patients. In ATTRv [128]. Many study described elevated NfL also in early stage of hATTR, showing a correlation with the severity of polyneuropathy, thus proposing NfL as a diagnostic and prognostic biomarker [10,129,130]. A further study confirmed the importance of NfL as a biomarker for nerve damage showing a significant decrease after therapy with Patisiran [131].

Ataxia with oculomotor apraxia is a group of recessive ataxias: ataxia-telangectasia (AT), ataxia with oculomotor apraxia type 1 (AOA1) and type 2 (AOA2). All these conditions share axonal neuropathy and elevated serum a.lpha-fetoprotein (AFP) [132]. AOA1 is an autosomal recessive disease due to mutations in the *APTX* gene, which encodes for aprataxin. During the course of AOA1, decreased serum albumin and increased serum total cholesterol can be seen [13,133]. In addition, hypoalbuminemia, hypercholesterolemia and increased serum creatine kinase (CK) were reported in association with increased AFP [134]. However, according to another study hypoalbuminemia and hypercholesterolemia with normal AFP are the hallmarks of AOA1 [135]. AOA2 is caused by mutations in the *SETX* gene, encoding for a DNA/RNA helicase protein [136]. Elevation of AFP is present in 99% of cases and is stable over the course of disease, but it usually do not correlate with disease severity [137]; elevated CK, mild hypoalbuminemia, or hypercholesterolemia can also be present in AOA2 patients [138]. Ataxia-telengectasia (AT) is caused by mutation in the *ATM* gene, that encodes a phosphatidylinositol-3 kinase protein (PI3K). Immunoglobulins (Ig) lare usually reduced in AT and elevation of AFP is progressive [138]. Conversely, in A-TLD, a rare form caused by mutations in the *hMRE11 gene*, patients have normal AFP serum concentrations [132,139].

Spinocerebellar ataxias (SCA) are a heterogenous group of diseases with CAG-trinucleotide expansions. Axonal polyneuropathy in SCA is one of the typical clinical features and depends on neuronal loss for inactivation of repair pathways [140]. Peripheral axonal neuropathy, tested by electrophysiology, is consistent among SCA1, 2, 3, 18, 25, 38, 43, 46 and specially SCA4 [5,141]. Neurofilament light polypeptide (NfL) is marker of neuronal damage. Serum concentrations of NfL are increased in patients with SCA, but the results have not already been validated [140,142].

Hereditary spastic paraplegias (HSP) are inherited neurodegenerative diseases, characterized by lower limbs weakness and spasticity [143]. Axonal neuropathy is frequent in SPG7, SPG11, and SPG15 and less commonly in SPG3A, SPG4, and SPG5 [143]. SPG5, a rare form of HSP, is caused by recessive mutation in the *CYP7B1* gene, encoding oxysterol-7α-hydroxylase [144]. CYP7B1 substrates, including 27-hydroxycholesterol (27-OHC), have been described cerebrospinal fluid (CSF) and in the sera of SPG5 patients; the latter were correlated with severity and duration of the disease [145].

Interestingly, serum 25-OHC and 27-OHC, have been validated as diagnostic biomarkers in SPG5 and it has been proved that atorvastatin can decrease serum 27-OHC by ∼30% [146].

Hepatic porphyrias (HP) are metabolic disorders of the heme metabolism; HP are caused by accumulation of heme intermediates due to enzymatic alteration in the heme biosynthesis pathway [147]. Acute intermittent porphyrias (AIP) is the most common subtype of HP and presents an autosomal dominant inherited pathology characterized by accumulation of porphyrin precursors, porphobilinogen (PBG) due to inherited deficiency of porphobilinogen deaminase [148]. Variegate porphyria (VP) is caused by deficiency of protoporphyrinogen oxidase (PPOX) that causes accumulation of δ -aminolevulinic acid (ALA) [147,148]. Porphyric attacks can be characterized by peripheral neuropathy, typically a motor neuropathy with symmetrical proximal muscle weakness [149]. Increased urinary porphobilinogen (PBG) and δ-aminolaevulinic acid (ALA) concentrations are usually detected during acute attacks, with normal interictal ranges; it has been reported that raised concentrations persist for many years in AIP patients [150].

GM2 gangliosidosis comprises Tay-Sachs disease, Sandhoff disease and GM2 gangliosidosis AB due to β-hexosaminidase A, β-hexosaminidase A+B and GM2 activator deficiency respectively. Several serums (i.e., lactic dehydrogenase malic dehydrogenase, fructose 1,6-diphosphate aldolase) and liquoral biomarkers (i.e., epithelial-derived neutrophil activating protein 78, monocyte chemotactic protein 1, macrophage inflammatory protein-1 alpha, macrophage inflammatory protein-1 beta, tumor necrosis factor receptor 2) were identified in gangliosidosis affected patients [151]. A recent study proposed serum aspartate transaminase (AST) as new potentially biomarker for gangliosidosis [151].

X-linked adrenoleukodystrophy (XALD) is the most common peroxisomal disorder due to mutations of the ABCD1 gene leading to very long-chain fatty acids (VLCFA) accumulation in blood and a variety of tissue [152]. In males with clinical suspicion, the diagnosis of XALD is allowed by elevated serum or plasma concentrations of hexacosanoic acid C26:0 and high ratio C24:0/C22:0 and C26:0/C22:0 [152]. An alternative and more accurate biomarker to detect the elevated serum VLCFA is the use of 1-hexacosanosyl-2-lyso-sn-3-glycero-phosphatidylcholine (26:0-lyso-PC) in DBS and it could be useful for newborn screening and diagnosis in women [153]. A recent study suggests new optimized cutoff values for both ratios C24:0/C22:0 and C26:0/C22:0, in combination with standard lipid profile considered that low-density lipid concentrations strongly correlate to all VLCFA [9].

Fabry disease (FD) is an X-linked inborn error of glycosphingolipid catabolism due to deficiency of α-galactosidase A (α-Gal A) leading accumulation of globotriaosylceramide in body fluids and lysosomes of vascular endothelium [154]. The diagnostic algorithm in FD is gender-specific indeed the measurement of blood α-Gal A activity is recommended in males, and optionally in females. Globotriaosysphingosine for identification of atypical FD variants and high- sensitive troponin T for identification of cardiac involvement are also important diagnostic biomarkers [6].

## 8. Demyelinating Neuropathies

Among this group of disorders, we report demyelinating CMT and inborn errors of metabolism.

### 8.1. Charcot–Marie–Tooth Spectrum Disorders

In the actual scenario there are no validated blood or liquoral biomarkers for CMT spectrum disorders, although a recent observational study suggested potentially new diagnostic and prognostic biomarkers in CMT1A patients [155]; indeed, the upregulation of the protein and lipid catabolism products plasma concentrations (i.e., dipeptide glutaminyl-serine, tryptophan, urobilinogen, polyamine acetylagmatine, sphingosine-1-phosphate, 6-hydroxysphingosine, lysophosphatidylcholine, 11-hydroxyeicosatetraenoate glyceryl ester) and downregulation of leucine plasma concentrations have been reported [155]. A more recent cross-sectional study showed the higher concentration of plasma neurofilament light chain in CMT patients correlating with disease severity [121]. In addition, it has been documented that some CMT1A patients can display serum PMP22 antibody and CSF protein content moderately elevated [156].

### 8.2. Inborn Errors of Metabolism

Inborn errors of metabolism (IEMs) are genetic disorders that cause disruption of a metabolic pathway leading to the accumulation of a toxic substrate [157]. IEMs may present as an apparently isolated peripheral neuropathy at any age or a polyneuropathy may be the only part of the clinical picture.

Refsum disease is an inborn error of lipid metabolism due to low phytanoyl-CoA hydroxylase activity leading to high phytanic acid plasma concentrations which represents the pathognomonic biomarker [158]. A relapsing-remitting polyneuropathy is the main feature with acute exacerbation in the setting of elevated phytanic acid blood concentrations caused by high phytanic acid intake, weight loss or pregnancy with elevated CSF protein concentration [159].

Tangier disease (TD) is an inherited lipid trafficking disorder characterized by severe deficiency or absence of high-density lipoprotein (HDL) in the circulation resulting in tissue accumulation of cholesteryl esters, particularly in the reticuloendothelial system with hyperplastic yellow-orange tonsil and hepatosplenomegaly [160]. As the high clinical suspicion, serum hypertriglyceridemia, very low HDL cholesterol and undetectable apolipoprotein A1 are pathognomonic for TD [161].

Metachromatic leukodystrophy (MLD) is caused by deficiency of lysosomal arylsulfatase A with cerebroside deficiency and sulfatides accumulation in various tissues. The diagnosis of MLD is confirmed by demonstrating deficient ASA activity in leukocytes and increased urinary sulfatide concentrations [162]. Some authors documented elevations of proinflammatory cytokines (e.g., IL8, IL-1Ra) and vascular endothelial growth factor in both CSF and plasma of MLD patients [163].

Krabbe disease (KD), also known as globoid cell leukodystrophy, is a rare lysosomal storage disorder caused by deficiency of galactocerebrosidase (GALC) leading to generation of psychosine, a cytotoxic agent that causes loss of oligodendrocytes and Schwann cells [164]. Newborn screenings are available for Krabbe disease with measurement of GALC enzymatic activity in DBS and measurement of psychosine in DBS as second-tier test may help to differentiate infantile from late-onset KD variants, as well as from GALC variant and pseudodeficiency carriers [165]. Psychosine concentrations have also been evaluated as biomarker to predict the severity of disease considering patients with psychosine greater concentrations at high risk to develop early-onset KD [166].

Zellweger spectrum disorders are caused by mutation in one of 13 different PEX genes resulting in different clinical phenotypes [167]. The higher plasma concentrations of very long-chain fatty acids (e.g., C26:0 lysophosphatidylcholine), polyunsaturated fatty acids, bile acid intermediates (i.e., di- and trihydroxycholestanoic acid), phytanic acid, pristanic acid, pipecolic acid and detectable urinary concentrations of bile acid intermediates or detectable pipecolic, glycolic, and oxalic acid may support the diagnosis [168].

Niemann–Pick disease is an autosomal recessive lipid storage disorder that comprises types A (NP-A) and B (NP-B) caused by deficiency of acid sphingomyelinase and type C (NP-C) caused by protein NPC1 defect leading to intracellular sphingomyelin and free cholesterol accumulation respectively. Reduced sphingomyelinase activity in circulating leukocytes, elevated both triglycerides and serum LDL-cholesterol and reduced HDL-cholesterol serum concentrations are the diagnostic hallmarks of the NP-B [169]. Cholesterol oxidation products such as 3β,5α,6β-triol and 7-ketocholesterol (7-KC) might represent a specific and sensitive blood-based biomarker for NP-C [170]. A further study identified two secreted proteins (i.e., galectin-3, cathepsin D) with increased serum concentrations in NPC1 patients correlating with disease severity [171]. Additionally, a recent study showed significantly decreased values of HSP70 in individuals with NP-C [172]. The combination of 7-KC, lysosphingomyelin and bile acid-408 improves the accuracy of NP-C diagnosis [173].

Abetalipoproteinemia typically presents in infancy with failure to thrive and malabsorption of fat and fat-soluble vitamins leading to multiorgan and neuromuscular involvement with cranial and peripheral nerve demyelination due to vitamin E deficiency. Common serums findings are extremely low LDL-cholesterol, triglyceride and apoB concentrations [174].

Beta-Mannosidosis is a lysosomal storage disease due to an isolated deficiency of the enzyme betas-mannosidase with various degrees of neurological deterioration. The diagnosis is supported by reduced enzyme activity in plasma and leukocyte [175]. Liquid chromatography-tandem mass spectrometry (UPLC-MS/MS) for urinary free oligosaccharides is a useful diagnostic tool [176].

Type 1 hyperoxaluria (PH1) is a rare autosomal recessive disease due to mutation in the *alanine-glyoxalate aminotransferase* gene leading to glyoxylate accumulation and its conversion to oxalate with calcium oxalate crystals deposition in various tissues [177]. The biochemical hallmarks of PH1 are hyperoxaluria with or without hyperglycolaturia; hence, 24 h urine collection measuring oxalate, creatinine and glycolate is recommended. Plasma oxalate concentrations are also useful in patients with renal failure, whereas higher ones are helpful to the diagnosis [12].

Tyrosinaemia type 1 (HT1) is a rare autosomal recessive disorder of amino acid metabolism resulting from fumarylacetoacetase 1 deficiency. The diagnosis is supported by increased plasma concentrations of tyrosine, methionine and phenylalanine, increased concentrations of hepatic transaminases and plasma alfa- fetoprotein [178]. As hypertyrosinemia is caused by other conditions, the diagnosis is allowed by the identification of the pathognomonic and more sensitive and specific biomarker succinylacetone on blood or urine with different cut-off reported [179,180].

Methylmalonic aciduria and homocystinuria cblC type are the most frequent inborn error of vitamin B12 metabolism. The biochemical hallmarks are elevated plasma and urine concentrations of methylmalonic acid, cystathionine and homocysteine with hypomethioninemia, whereas metabolic acidosis and hyperammonemia have been rarely reported [181].

Cerebrotendinous xanthomatosis (CTX) is an autosomal recessive inborn lipid storage disorder due to enzyme sterol 27-hydroxylase deficiency resulting in impaired bile primary acid synthesis, increased concentration of bile alcohols and increased accumulation of both cholestanol and cholesterol in plasma and in various tissues, particularly in the nervous system [182]. High plasmatic concentrations of cholestanol in the presence of normal or low plasma concentrations of cholesterol, decreased chenodeoxycholic acid and increased concentration of bile alcohols are the laboratoristic hallmark of CTX [183].

## 9. Conclusions

Neuromuscular disorders can be very complex with the involvement of various organs and systems. A number of biomarkers are to date available to support the diagnosis of INMD, but the role of expert clinician is crucial. Some markers are commonly altered in many conditions in similar way in different disorders, so that the physical examination should be the first step to raise the clinical suspicion. The complete neuromuscular examination, followed by subsequent evaluations is the easiest way to obtain diagnoses to date. On the other hand, some biomarkers are associated with specific disorders and their recognition can be crucial for the diagnosis. Indeed, it is not unusual to see clinicians who are not familiar with rare diseases facing with INMD, because of the very broad range of serum and urinary alterations encountered in clinical practice. Hence, the complete knowledge of such abnormalities can accelerate the diagnostic workup supporting the referral to specialists in neuromuscular disorders.

## Data Availability

Data sharing not applicable. No new data were created or analyzed in this study. Data sharing is not applicable to this article.

## Supplementary Materials

The following are available online at https://www.mdpi.com/2076-3425/11/3/398/s1, Table S1: main biomarkers, Diagram S1: increase of serum CK.
