# Supplementary material for: Inherited Neuromuscular Disorders: Which Role for Serum Biomarkers?
Source: Brain Sci. 2021 Mar 21;11(3):398. doi: 10.3390/brainsci11030398 (PMC8004068; doi:10.3390/brainsci11030398)
Supplement: Supplementary file 1 [file brainsci-11-00398-s001.pdf]

Table S1. Main biomarkers.

Marker	Disease	Values	Persistent or Episodic	Triggers	Prognostic Correlation	Differential Diagnosis
25-OHC	SPG5	Increased	Persistent	//	negative	//
27-OHC	SPG5	Increased	Persistent	//	negative	//
3β,5α, 6β-triol	Niemann-Pick C	Increased	Persistent	//	Negative	Fabry disease<br>Gaucher disease<br>Mucopolysaccharidosis type IV<br>GM-1 gangliosidosis<br>GM-2 gangliosidosis
7-ketocholesterol	Niemann-Pick C	Increased	Persistent	//	Negative	Fabry disease<br>Gaucher disease<br>Mucopolysaccharidosis type IV<br>GM-1 gangliosidosis<br>GM-2 gangliosidosis
Acylcarnitine profile	MADD	Increased (All intermediates)	Persistent	Fasting, fever, drugs, physical exercise, emotional stress.	//	Extreme metabolic stress
	VLCAD	Increased (Very Long Chain intermediates)				
	SCAD	Increased (Short chain intermediates)				
AFP	AOA2	Increased	persistent	//	//	Cancer
	Type 1 tyrosinaemia	Increased				
Albumin	AOA1, AOA2	Decreased	persistent	//	//	Chronic medical conditions associated with cachexia
Anti-Müllerian hormone	DM1	Decreased	Persistent	//	Decreased ovarian reserve	//
Apolipoprotein A1	Tangier disease	Undetectable	Persistent	//	//	//
Apolipoprotein B	Abetalipoproteinemia	Extremely decreased or undetectable	Persistent	//	//	Homozygous hypobetalipoproteinemia
Bile acid 408	Niemann-Pick C	Increased			//	
Bile alcohols	Cerebrotendinous xanthomatosis	Increased	Persistent	//	//	//
BPN	ATTR(V122I)	Increased	Persistent	//	Heart failure	Other causes of heart failure
C26:0 lysophosphatidylcholine	X-Adrenoleukodystrophy<br>Zellweger spectrum disorders	Increased	Persistent	//	//	//
Carbonic anhydrase III	FSHD	Increased	persistent	//	Disease progression	//
Carnitine	Primary carnitine deficit	Extremely decreased	Persistent	//	//	//
	Secondary carnitine deficit (MADD)	Decreased	Episodic	//	//	//
Cathepsin D	Niemann-Pick C-1	Increased	Persistent	//	Negative	Lysosomal storage disorders (e.g., Gaucher disease, GM1 and GM2 gangliosidosis)

Cholestane-triol	Niemann-Pick C	Increased	Persistent	//	Negative	
Cholestanol	Cerebrotendinous xanthomatosis	Increased	Persistent	//	//	//
cholesterol	AOA1, AOA2	increased	Persistent	//	//	Dyslipidaemias
Citrulline	DMD	Primary carnitine deficit	Persistent	//	//	//
Crn	SMA 1-2-3	Decreased significant differences with SMA type SMA3>SMA2>SMA1	Persistent reduction of serum Crn markers	//	Phenotypic severity: walkers> sitters> non sitters. Severity of denervation (distal CMAP amplitude and MUNE)	chronic medical conditions associated with cachexia
	DMD—BMD - LGMD2A—LGMD2B.	Decreased	Persistent	//	disease progression	//
CTGF	Pompe Disease	Increased	Persistent	//	//	Muscular dystrophies
Cytolysis indexes*	All conditions With increase of CK	Increased	Persistent and episodic	//	correlation with renal failure.	//
Deoxyuridine	Mitochondrial neurogastrointestinal encephalopathy	Increased	Persistent	//	//	//
Dihydroxycholestanoic acid	Zellweger spectrum disorders	Increased	Persistent	//	Negative	α-methylacyl-CoA racemase deficiency
Fibronectin	DMD	Increased	Persistent	//	//	//
Galectin-3	Niemann-Pick C-1	Increased	Persistent	//	Negative	Lysosomal storage disorders (e.g., Gaucher disease, GM1 and GM2 gangliosidosis)
Globotriaosylceramide (Gb3)	Fabry disease	Increased	Persistent	//	//	//
HDL cholesterol	Tangier disease	Decreased (more than Nieman-Pick B)	Persistent	//	//	//
	Niemann-Pick B	Decreased	Persistent	//	//	//
Ig	AT	Decreased	Persistent	//	//	Other causes of hypogammaglobulinemia
IL1Ra	Metachromatic leukodystrophy	Increased	Persistent	//	//	Systemic inflammation
IL-8	Metachromatic leukodystrophy	Increased	Persistent	//	//	Systemic inflammation
Kalemia	Hyperkalemic paralysis	Increased	Episodic	Potassium-rich food, rest after exercise, fasting, cold, emotional stress, pregnancy	//	//

	Hypokalemic paralysis	Decreased	Episodic	Glucidic meals, alcohol, rest after exercise	//	Thyrotoxic periodic paralysis
LDL cholesterol	Niemann-Pick B	Increased	Persistent	//	//	Metabolic syndrome Inherited dyslipidaemia
	Abetalipoproteinemia	Extremely Decreased	Persistent	//	//	//
Leucine	CMT1A	Decreased	Persistent	//	Negative	Malabsorption Fasting state Sarcopenia
Lyso-GB2	Atypical Fabry disease	Increased	Persistent	//	//	//
Lysosphingomyelin	Niemann-Pick C	Increased	Persistent		//	
Methylmalonic acid	Methylmalonic aciduria and homocystinuria	Increased	Persistent	Renal failure	//	//
NF-H	SMA	Increased	Persistent (values decreases with age)	//	pNF-H inversely correlate with age at first dose of nusinersen	Aspecific marker of axonal injury and neuronal degeneration
	ALS	Increased	Persistent	//	//	
NfL	CMT1A CMTX1 HSN1 CMT1B CMT2A CMT4B2 CMT4C	Increased	Persistent	//	negative	Aspecific marker of axonal injury and neuronal degeneration
	ATTR			//	negative	//
	SCA1,SCA3			//	//	//
NT-pro-BNP	ATTR	Increased	//	//	Heart failure	//
	DM1—DM2	Increased	//	//	Cardiac involvement with ECG abnormalities	
Oxalate	Type 1 Hyperoxaluria	Increased	Persistent	//	//	//
Oxalate/creatinine ratio	Type 1 Hyperoxaluria	Increased	Persistent	//	//	//
PDGF-AA	Pompe Disease	Increased	Persistent	//	//	Muscular dystrophies
PDGF-BB	Pompe Disease	Decreased	Persistent	//	Higher in asymptomatic	
Phytanic acid	Refsum disease	Increased	Persistent	Pregnancy Weight loss Food (i.e., lamb, fish, beef, dairy products)	//	Other peroxisomal disorders
	Zellweger spectrum disorders	Decreased				
Pipecolic acid	Zellweger spectrum disorders	Increased	Persistent	//	Negative	
PMP22 antibody	CMT1A CMT2	Increased (normally undetectable)	Persistent	//	//	//
Pristanic acid	Zellweger spectrum disorders	Increased	Persistent	//	//	//

Proteic and lipidic catabolites**	CMT1A	Increased	Persistent	//	Negative	Fasting state
Psychosine	Krabbe disease	Increased	Persistent	//	//	//
RBP4	ATTR(V122I)	Decreased	Persistent	//	negative	//
Serum Lactate (forearm test)	MCardle Glycolysis deficit	Not Increased after ischemic and non-ischemic test	//	//	//	//
Serum Lactate (aerobic exercise)	Mithocondrial myopathies	Increased after aerobic exercise	Episodic	//	//	//
Serum Lactate (basal value)	Mithochondrial Myophaties.	Increased	Persistent	//	//	Severe infections, IR
TGF B2	Limb-Girdle muscular Dystrophies	Increased	Persistent	//	//	//
TGF-β	Pompe Disease	Increased	Persistent	//	//	Muscular dystrophies
Thymidine	Mitochondrial neurogastrointestinal encephalopathy	Increased	Persistent	//	//	//
Total serum adiponectin	DM1	Decreased	Persistent	//	worsening of IR	//
TrI	ATTR(V122I)<br>FSHD	Increased	Persistent	//	Heart failure	Other causes of heart failure
Triglycerides	Tangier disease	Increased	Persistent	//	//	Metabolic Syndrome Inherited dyslipidaemia
	Niemann-Pick B	Increased	Persistent	//	//	
	Abetalipoproteinemia	Extremely decreased or absent	Persistent	//	//	Homozygous hypobetalipoproteinemi a
	DM1	Increased	Persistent	//	//	//
Trihydroxycholestanoi c acid	Zellweger spectrum disorders	Increased	Persistent	//	Negative	//
TrT	ATTR(V122I)<br>DM1, DM2<br>Fabry disease	Increased	Persistent	//	Heart failure	Other causes of heart failure
tumor necrosis factor a	DM1	Decreased	Persistent	//	Worsening of insuline resistence (IR)	//
Tyrosine	Type 1 tyrosnaemia	Increased	Persistent	//	//	Benign newborn hypertyrosinemia Premature babies, sick babies on total parenteral nutrition, mitochondrial depletion syndromes, other metabolic or non-metabolic liver diseases (e.g., tyrosinemia types II and III)
Very long-chain fatty acid	X-Adrenoleukodystroph y	Increased C24:0/C22:0 >1 C26:0/C22:0 >0,02	Persistent	//	//	Ketogenic diet, hemolyzed samples, peanut rich diet
	Zellweger spectrum disorders	Increased C26:0/C22:0 >0,02				
Vitamin B 6	MCardle disease	Extremely Decreased (only mutation R49X)	Persistent	//	//	B6 deficiency

*Cytolysis indexes includes (CK isoforms, transaminases lactate dehydrogenase, myoglobin).

** Proteic and lipidic catbolites dipeptide glutaminyl-serine, tryptophan, urobilinogen, polyamine acetylagmatine, sphingosine-1-phosphate)

Neurofilamenti light chain (NfL), Charcot–Marie–Tooth (CMT), hereditary amyloidogenic transthyretin (hATTRv), serum retinol-binding protein 4 (RBP4); B-type natriuretic peptide (BNP), transiterin (TTR), Troponin I (TrI), amino-terminal prohormone of brain natriuretic peptide (NT-proBNP); Troponin T (TrT), ataxia-telangectasia (AT), oculomotor apraxia type 1/2(AOA1/2), alpha-fetoprotein (AFP), creatine kinase (CK), immunoglobulin (Ig); 27-hydroxycholesterol (27-OHC); porphobilinogen (PBG); δ-aminolaevulinic acid (ALA), malignant hyperthermia syndrome (MMS), hypokalemic paralysis (HypoPP), hyperkalemic paralysis (HypePP), facioscapolohumeral distrophy (FSHD), multiple acylcoa dehydrogenases deficiency (MADD), very long chain acilcoa dehidrogenase deficiency (VLCAD), short chain acilcoa dehidrogenase deficiency (SCAD), creatine kinase (CK), heavy chain neurofilaments (NF-H), creatinine (pNF-H), compound muscle action potential (CMAP); motor unit number estimation (MUNE), myotonic dystrophia 1 (DM 1), Duchenne muscular dystrhophia (DMD).

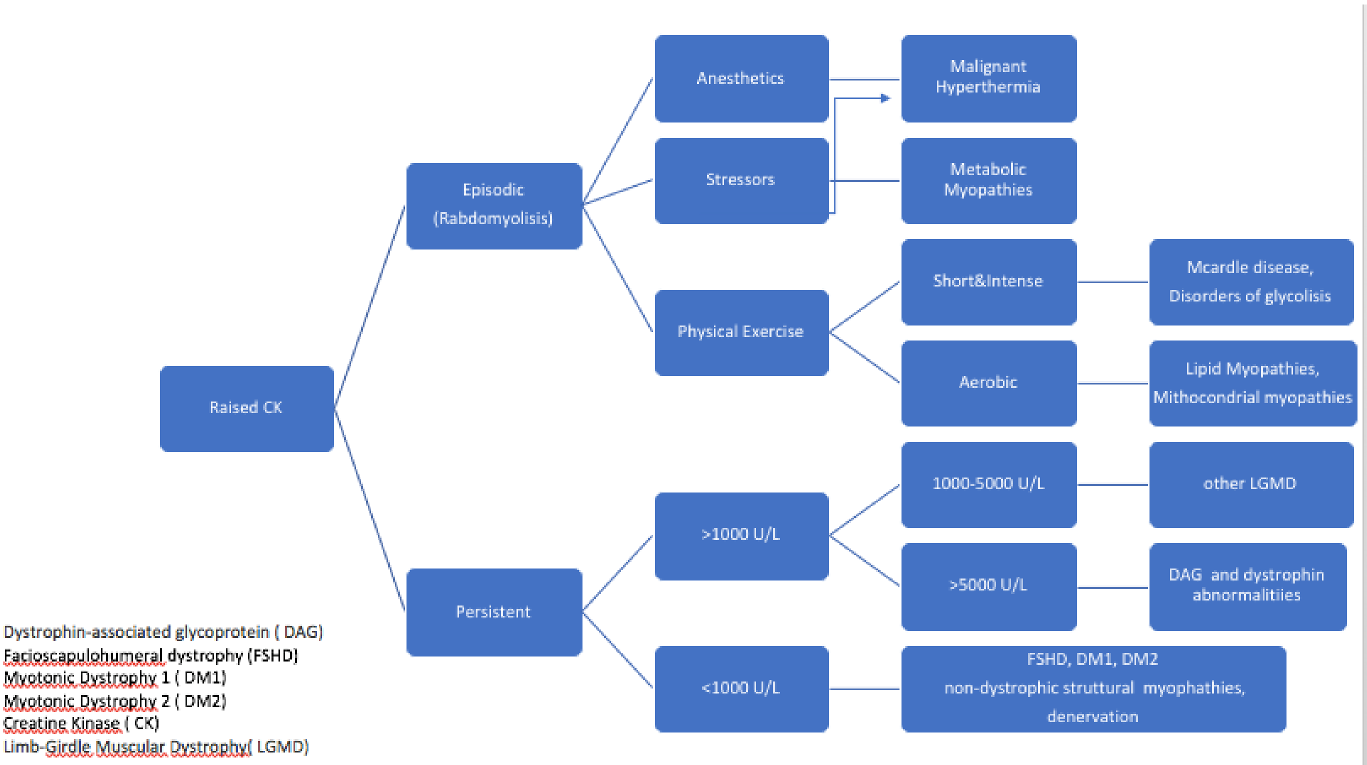

Diagram S1: Increase of serum creatine kinase (CK).
